# What Cures? and by What Method?

**Published:** 1892-04

**Authors:** B. L. B. Baylies

**Affiliations:** Brooklyn, N. Y.


### WHAT CURES? AND BY WHAT METHOD?

B. L. B. Baylies, M. D., Brooklyn, N. Y.

(Read before the Kings County Homœopathic Medical Society, October, 1891.)

It almost seems the prevalent idea that matter is the healing agent. It is not matter in the ordinary sense, but the peculiar forces inherent in and evolvable from matter which cure. Careful observers find that homœopathic medicine, whether given high or low as to "potency," if too long repeated, aggravates the symptoms extant, and develops others; so that it becomes necessary to suspend its use, and allow the medicinally aroused and directed vital-force to accomplish the cure. We admit the disastrous complication of disease produced by long administration of the crude drug; for example, the complication of syphilis by excessive dosing with Mercury; and recognizing that susceptibility to the similar medicine is heightened by disease, verify by observation our surmise, that the most minute or infinitesimal dose if too frequently repeated, complicates and renders more inveterate the morbid disorder, sustaining the precept of Hahnemann, "Omit medicine when improvement begins, and desist from medicine while it continues."

No disease has to me appeared more demonstrative of the influence of medicine, and of the value of the most minute doses, than intermittent fever. The tendency in any case of this disease under unchanged local conditions is to retain its peculiar symptoms until affected by medicine; but it has been observed, when an unsuitable medicine, China, for instance, was given, even at the 200th, the succeeding paroxysm was imbued and modified by the symptoms of China, and its characteristics being changed or adulterated, the selection of the remedy was rendered more difficult; on the other hand, when a single minimal dose of the similar medicine was given, the following attack became milder, and usually all morbid phenomena ceased with the third paroxysm. The single dose was most beneficial, while the effect of repeated doses, even of the homœopathic remedy, especially in low potencies, protracted the case and discouraged both physician and patient; the aggravation produced by its repetition obscured its homœopathic action, and here we see the unwisdom which may lead the discouraged from abused Homœopathy into pernicious allopathy. It is fair to draw a practical lesson from such experience and to apply it in the treatment of all diseases, viz.: to employ the single similar medicine, the least requisite dose, with the least requisite repetition.

The following case is not presented as a typical illustration of the preceding remarks, but an endeavor to *approximate* the principles therein stated:

### SPINAL CONGESTION OR SPINAL MYELITIS.

Antonia S., aged nineteen, dark haired, of sanguine temperament, generally healthy, had for ten days pain the whole length of the spine, and on both sides of it, from its lower extremity to the base of the cranium, with great sensitiveness to pressure.

When bending forward the pain extends from the lower back upward. She has frequent "rushes of blood to the head," the face feels hot and has a jacqueminot flush. She had occipital headache and sensation of fullness or congestion of the head, with stiffness of the nape of the neck on turning it, pain shooting from the shoulder down the right arm to below the elbow, with a continual stiffness and aching, worse when raising the arm to the head; also a sensation of "choking tightness," as she expresses it, in the middle of the sternum, and sharp pains through and from the sacro-lumbar region down the back of lower extremeties to below the knees. Iron presents the symptoms given, tearing from the top of the right shoulder down the upper arm, causing inability to raise it, is one of its characteristics; the direction of the spinal pain upward, *except as to the nape of the neck*, is not mentioned in the proving.

℞ May 23d, 1890, Ferrum-met.$^{45m}$ (Fincke). One dose.

May 27th, head entirely relived of pain and fullness; the upper arm relieved; no pain or stiffness of the neck; pain still extends from the lowest back upward to the infra-scapular regions; the sacro-lumbar portion still painful, though with the pains extending from it down the lower limbs, less severe. No medicine.

May 29th. Facial paralysis has appeared on the right side. ℞ Thuja$^{5m}$ (F.), one dose, dry.

June 2d. Face somewhat drawn to the left, moderately flushed, no headache, the whole back painless, numb; legs heavy and draggy from the knees down; ascending difficult. She habitually now, as before this sickness, lies upon the back; both sides feel sore when lain upon, though she feels less uneasy upon the right side. The menses appear at intervals of from three to four months, the last occurring a month ago, painless, the flow scanty and pale; face flushed in proportion to delay of menses; the hands and feet burn, especially the soles; indigestion; wants to be uncovered in bed, but soon wants to be covered again. June 2d, Sulphur$^{m}$ (F.).

June 10th. Facial paralysis nearly obliterated; she is able to close both eyes; head and stomach "all right." A steady ache has appeared between the scapulæ; when lying, soreness upon the side lain upon, corresponding to the lower ribs; feet still heavy when walking, more when ascending, numb while sitting, face flushed. ℞ Puls.$^{45m}$ (F.), a dose. Pulsatilla is worse lying on either side, especially the left; has the symptom, wanting to uncover from heat, but soon chilly, wanting to be covered; and possibly it should have been given instead of the Sulphur, though improvement followed the latter.

June 17th. Face free from paralysis; interscapular pain now only present in the morning, except slight return of it in the evening; she sleeps well, still lying on the back; occasionally, since the Pulsatilla, lies on the right side; a little soreness when on the left side; feels less heavy; very slightly numb while sitting.

June 30th. She feels entirely well, except a slight, interscapular pain on awaking in the night or morning, which passes off by change of position or rising from bed; lower extremities without numbness or heaviness. It is now (June 30th) two months since the last menses. ℞ Pulsatilla$^{m}$ (F.), one dose, dry.

July 7th. Normal menstruation during the last week. She is quite well, has continued to be well until the present writing, December 2d, 1891.

Duration of treatment, from May 23d to July 7th.

